# Unraveling Spinal Cord Injury Nutrition: Effects of Diet on the Host and Microbiome

**DOI:** 10.1016/j.advnut.2025.100448

**Published:** 2025-05-16

**Authors:** ZeHui Li, XiaoXin Wang, HuaYong Du, WuBo Liu, ChunJia Zhang, Zuliyaer Talifu, Xin Xu, Yunzhu Pan, JinMing Zhang, Han Ke, DeGang Yang, Feng Gao, Yan Yu, YingLi Jing, JianJun Li

**Affiliations:** 1 School of Rehabilitation, Capital Medical University, Beijing, People's Republic of China; 2 Department of Spinal and Neural Functional Reconstruction, China Rehabilitation Research Center, Beijing, People's Republic of China; 3 China Rehabilitation Science Institute, Beijing, People's Republic of China; 4 Department of Orthopaedics, Qilu Hospital, Cheeloo College of Medicine, Shandong University, Shandong, People's Republic of China; 5 Department of Rehabilitation Medicine, Peking University Third Hospital, Beijing, People's Republic of China; 6 School of Population Medicine and Public Health, Chinese Academy of Medical Sciences/Peking Union Medical College, Beijing, People's Republic of China; 7 University of Health and Rehabilitation Sciences, Shandong, People's Republic of China; 8 Rehabilitation Department, Beijing Hospital, National Center of Gerontology, Institute of Geriatric Medicine, Chinese Academy of Medical Sciences, Beijing, People's Republic of China; 9 Department of Orthopedics, Beijing Chaoyang Hospital, Capital Medical University, Beijing, People's Republic of China

**Keywords:** spinal cord injury, nutrition, gut microbiota, diet, metabolic alterations, personalized nutrition management

## Abstract

Spinal cord injury (SCI) leads to severe neurological dysfunction with significant nutritional alterations. These alterations are closely associated with gut dysbiosis and neurogenic gut dysfunction after SCI, creating complex interactions that further exacerbate metabolic disturbances and impede neurological recovery. In the context of SCI, diet not only fulfills basic nutritional needs but also serves as an important therapeutic tool to modulate these interactions. This review provides a broad overview of existing research findings, analyzes the impact of existing dietary interventions on SCI, and attempts to clarify the complex relationship between diet and host and gut microbiota. We hope to provide a clear direction for future research and a scientific basis for the development of personalized dietary interventions to improve the nutritional status of patients with SCI, reduce the incidence of complications such as metabolic disorders, and promote the recovery of neurological function and overall quality of life of patients with SCI.

## Graphical abstract

**Figure:**
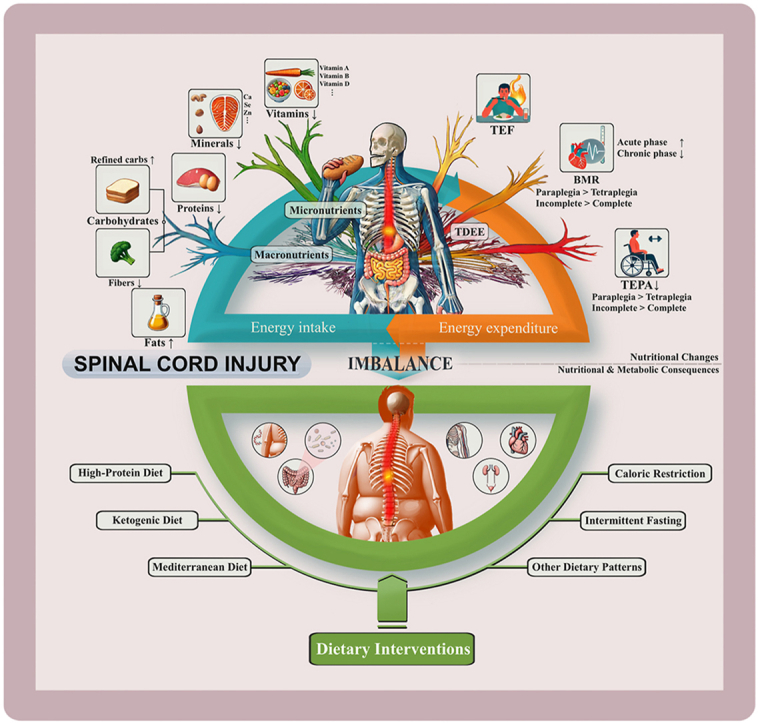

**Statement of significance**

This review evaluates the nutritional changes in patients with spinal cord injury (SCI), comprehensively elucidating the effects of dietary interventions on patients with SCI from both the host and gut microbiota perspectives. By revealing the complex interactions among them, it lays the foundation for developing personalized nutritional intervention strategies to optimize recovery and improve long-term health outcomes in the future.

## Introduction

**FIGURE 1:**
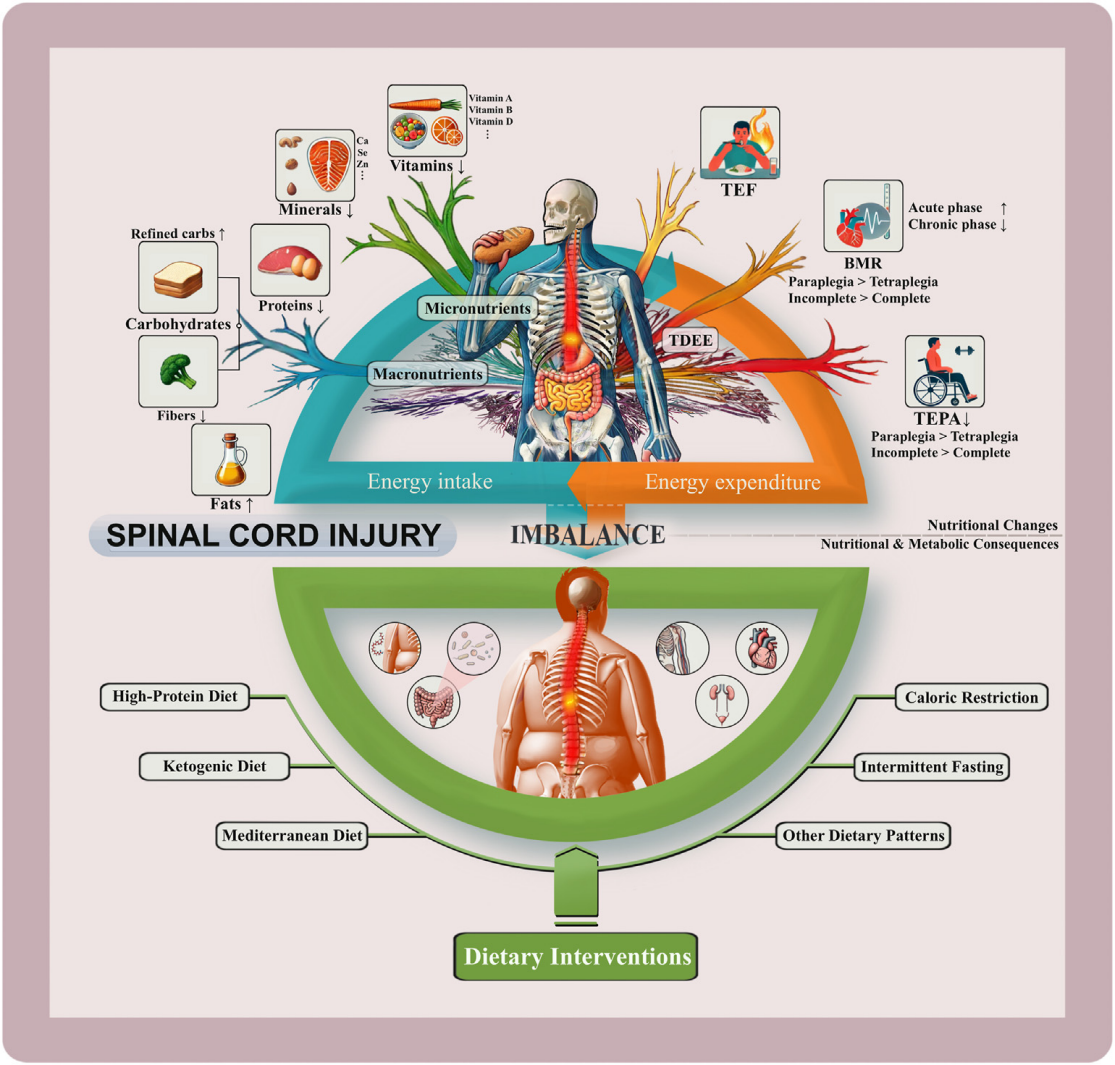
Impact of nutritional changes and energy imbalance on systemic health post-SCI. The figure illustrates the impact of nutritional changes after SCI on overall health, highlighting potential improvements through various dietary interventions. The upper left shows changes in micronutrient and macronutrient intake after SCI, which subsequently alter energy intake. The upper right shows changes in total daily energy expenditure (TDEE) including basal metabolic rate (BMR), thermic effect of activity (TEA), and thermic effect of physical activity (TEPA). Changes in energy balance and nutrient intake after SCI lead to nutritional and metabolic consequences impacting multiple organ systems in the lower part. Additionally, the lower section presents dietary interventions, such as HPD, KD, MD, IF, CR, and other dietary patterns, which may influence health regulation in patients with SCI through various mechanisms. Arrows indicate trends: “↑” for increases and “↓” for decreases. CR, caloric restriction; HPD, high-protein diet; IF, intermittent fasting; KD, ketogenic diet; MD, Mediterranean diet; SCI, spinal cord injury.

Spinal cord injury (SCI) is a devastating condition that leads not only to severe neurological impairment but also to significant nutritional changes [1]. These imbalances commonly include an energy intake that exceeds daily energy expenditure, which can vary across different injury stages and types [[2], [3], [4], [5]]. In addition, SCI-related dietary patterns are often high in simple carbohydrates and fats, and low in fiber, with concurrent deficiencies in a range of micronutrients [[4], [5], [6], [7]] (Figure 1 for specific variations). Such nutritional problems are closely linked to the complex physiological and psychosocial factors of SCI, including reduced energy needs, increased inflammation, and physical limitations, alongside **s**ocial changes and emotional challenges [[8], [9], [10], [11], [12], [13], [14], [15]]. Moreover, these challenges are influenced by individual factors such as the severity, duration, and level of the injury [[16], [17], [18]]. As a result, the nutritional issues faced by patients with SCI differ substantially from those seen in the general population.

Studies show that dietary adjustments can directly influence patient outcomes by achieving a more favorable energy balance and nutrient profiles, as well as indirectly through effects on the “microbiota-gut-brain axis,” mediated by gut microbiota and their metabolites [3,[19], [20], [21]].

Changes in gut microbiota further complicate the nutritional landscape after SCI [22]. The gut microbiota undergoes significant alterations in the acute phase of injury, which persist and correlate with injury severity and associated complications [20,23]. These changes often hinder neurological recovery [20,24]. In addition, shifts in the gut microbiota can increase intestinal permeability, affecting the absorption of important nutrients (e.g. zinc, selenium) and disrupting normal metabolic processes [[25], [26], [27]].

This review examines how specific nutrients and overall dietary patterns influence recovery after SCI, and explores the role of gut microbiota in these processes. By understanding these complex relationships, we can lay the groundwork for tailored nutritional strategies that optimize recovery and long-term health for individuals living with SCI.

## Impact of Diet on SCI

In SCI, nutrition can serve as more than basic sustenance—it can be a therapeutic tool to shape physiological and metabolic responses after injury. This section examines how specific nutrients and dietary patterns influence SCI recovery and explores possible underlying mechanisms.

### The effects of specific nutrients on SCI

#### Omega-3 PUFAs in SCI

Omega-3 PUFAs (ω-3 PUFAs), including Eicosapentaenoic Acid (EPA), docosahexaenoic acid (DHA), and alpha-linolenic acid, are essential fatty acids known for their anti-inflammatory and neuroprotective effects [28]. In SCI, patients often display abnormal PUFA metabolism, especially involving omega-6 fatty acids and marked DHA deficiency [29].

Rodent studies suggest that omega-3 PUFAs may aid in neural repair and promote functional improvements after SCI. For example, rats with SCI fed diets rich in omega-3 PUFAs showed increased motor scores and enhanced sensory test performance, regained voluntary bladder control, and experienced reduced neuropathic pain. These benefits persisted for ≥8 wk post injury and were linked to decreased expression of pain-related markers like Calcitonin Gene-Related Peptide (CGRP) and Mitogen-Activated Protein Kinase (MAPK) [29]. Omega-3 supplementation also outperformed other fatty acids in promoting functional recovery in mice after SCI [30], partly by improving the survival of neurons and oligodendrocytes and modulating immune cell activity [30,31]. In other animal studies, omega-3 PUFAs stimulated macrophage-mediated debris clearance and increased the expression of repair proteins [Myelin Basic Protein (MBP), Galc, Glial Fibrillary Acidic Protein (GFAP)] through inhibition of the mTORC1 pathway [32]. Moreover, FABP5 appears to facilitate DHA uptake, further supporting nerve regeneration in SCI rat model [33].

Combining DHA with curcumin increased the expression levels of learning-related molecular markers [Brain-Derived Neurotrophic Factor (BDNF), cAMP Response Element-Binding protein (CREB), Ca²⁺/Calmodulin-dependent Protein Kinase II (CaMKII), Syntaxin-3] and decreased oxidative stress indicators 4-Hydroxy-Nonen a l (4-HNE) in mouse and rat models [34,35]. Alpha-lipoic acid, another antioxidant, was associated with enhanced metabolic parameters (e.g. improved insulin sensitivity, or reduced fasting glucose) in males with chronic SCI when combined with reduced fat and carbohydrate intake [36,37].

Although these findings are promising, large-scale randomized controlled trials in humans are lacking. More clinical research is needed to confirm the potential therapeutic benefits of omega-3 PUFA supplementation in SCI.

#### Micronutrients in SCI

Patients with chronic SCI frequently experience imbalances in vitamins and minerals. A 2018 meta-analysis found that they often lack vitamins A, B5, B7, B9, D, E, as well as potassium and calcium, whereas intakes of vitamins B1, B2, B3, B12, C, K, and minerals like sodium, phosphorus, copper, and zinc exceed recommended values [4]. Vitamin D deficiency is particularly common, influenced by reduced physical activity, altered eating patterns, and limited outdoor time—even among elite SCI athletes [38,39]. Prolonged vitamin D deficiency increases the risk of bone loss and fractures [40].

Vitamins can play important roles in immune regulation, antioxidant defense, and neural repair. Vitamin C reduces oxidative stress and kidney damage in SCI rats by inhibiting NF-κB signaling [41]. Vitamin D helps maintain the intestinal barrier and influences gut microbiota composition in SCI rat models, reducing harmful bacterial entry and inflammation [42,43]. Vitamin E promotes oligodendrocyte proliferation, alleviates H-reflex inhibition, and supports bladder and motor function in rats with SCI [44]. The vitamin A metabolite all-trans retinoic acid supports axonal repair and motor recovery [45,46], and vitamin D may reduce neuronal apoptosis while encouraging axonal and myelin regeneration in both animal models and clinical studies [43,47]. B vitamins help restore nerve function by reducing endoplasmic reticulum stress and apoptosis, thus improving neuronal survival and conduction [48,49].

Minerals also matter. In SCI rat models, moderate zinc intake reduces oxidative stress and supports neurotrophic factors like BDNF, aiding nerve repair [50]. Selenium, an antioxidant enzyme component, helps restore bladder function of SCI rats, though it shows limited impact on motor function [51]. Addressing mineral imbalances, such as those involving calcium, can help maintain bone mineral density and lower the risk of fractures common in patients with SCI [[52], [53], [54]].

Balancing vitamin and mineral intake can enhance antioxidant defenses, support nerve repair, and may improve long-term rehabilitation in patients with SCI. Although current public health guidelines recommend supplementation only in specific cases [55], targeted supplementation may benefit those with suboptimal postinjury nutrition [56,57]. Individualized nutritional plans that consider these micronutrient needs are essential for optimizing rehabilitation outcomes.

#### Functional foods and other neuroprotective substances in SCI

Growing evidence points to the potential of functional foods and neuroprotective compounds in SCI management. Such interventions may offer direct nervous system protection or indirectly influence recovery through the gut microbiota and metabolic pathways.

Kefir, a probiotic-rich fermented dairy product, has shown neuroprotective effects in animal models by reducing neuronal damage, lowering oxidative stress, and enhancing gut health of SCI rats during spinal cord ischemia/reperfusion injury [58]. Limonene and its metabolite perillyl alcohol enhance nerve regeneration, enhance motor function, and reduce pain sensitivity in SCI mice models, partly by increasing the expression of growth-associated proteins and nerve growth factors [59]. Although limonene occurs naturally in citrus fruits, therapeutic doses typically require supplementation.

Green tea extract, rich in bioactive compounds like catechins, lowers liver iron accumulation and blood glucose levels in patients with SCI [60]. Although green tea extract may not fully prevent increases in liver fat or inflammation, continued intake before and after injury could offer partial liver protection.

Overall, these functional foods and neuroprotective substances hold promise as complementary strategies in SCI rehabilitation. Future research should clarify their mechanisms, determine optimal doses, and explore their incorporation into comprehensive treatment plans.

### Impact of dietary patterns on SCI

Although examining the effects of specific nutrients provides valuable insights, understanding overall dietary patterns offers a more accurate picture of how daily eating habits influence SCI recovery [19]. Similarly, recent American dietary guidelines place greater emphasis on overall dietary patterns rather than focusing solely on individual micro- and macronutrients [7,61]. Dietary patterns, defined by variations in nutrient ratios, energy intake, and specific nutritional components, can affect the recovery process of SCI to different extents through multiple mechanisms (Figure 1). Next, we will discuss common dietary patterns that are currently the focus of research (Table 1) [40,[62], [63], [64], [65], [66], [67], [68], [69], [70], [71], [72], [73], [74], [75], [76], [77], [78], [79], [80], [81], [82], [83], [84], [85], [86], [87], [88], [89], [90], [91], [92], [93], [94], [95], [96], [97], [98], [99]].

**TABLE 1:** Dietary patterns and their impact on gut microbiota and spinal cord injury recovery.

Reference	Model (sex/age/weight/AIS grade/SCI phase)	Sample size	SCI method (animal model/clinical injury type)	SCI damaged segment	Dietary intervention			Major finding
					Dietary pattern	Macronutrient ratio	Intervention protocol	
Aynur Demirel et al., 2020 [62]	Human (18–60 y; AIS A-C, acute)	*N* = 60	Incomplete	C5-T12	KD	3:1 fat:carbohydrates + protein	5 wk	Ongoing study; previous pilot study showed improved upper extremity motor function and reduced serum levels of a neuroinflammatory blood protein.
Bo-Tao Tan et al., 2020 [63]	SD rats (male, 3–4 mo, ∼350 g)	*N* = 49	Hemicontusion	C5	KD + KS	KD: F5848; paste; Bio-Serv KS: calcium/sodium βHB mineral salt; 1000–1500 mg βHB; sold under the trade name KetoCaNa by KetoSports; intragastric administration	KD: starting at 3 h post injury for 8 wk KS: delivered at 12-h intervals for 4 d post injury	Rapid increase in blood ketone levels; improved forelimb motor performance; increased neuronal and axonal survival in the dorsal corticospinal tract; no significant effect on white matter sparing.
Hong Zeng et al., 2021 [64]	SD rats (male, 8 wk)	*N* = 15	Hemicontusion	C7	KD	4:1 fat:carbohydrates + protein	70 g/kg body weight/d for 4 wk	Improved myelin growth; significant improvement in steroid anabolic pathway; reduced expression of genes in immune-related pathways.
Kong et al., 2021 [65]	SD rats (male, 275–325 g)	*N* = 52	Hemicontusion	C5	KD	3.1:1 fat:carbohydrates + protein	2 wk pre-SCI	Inhibited NLRP3 inflammasome and shifted macrophages/microglia from M1 to M2a phenotype; Increased Arginase 1, decreased CD16 and iNOS; improved functional recovery.
Liu et al., 2018 [66]	SD rats (male, 3–4 mo, 250–300 g)	*N* = 32	Posterolateral lumbar spinal fusion	L4/5	KD	4:1 fat:carbohydrates + protein	8 wk	Delayed spinal fusion; decreased bone mass at fusion sites; reduced IGF-1 levels; no significant difference in serum calcium or phosphorus levels.
Lu et al., 2018 [67]	SD rats (male, 8 wk, 300–360 g)	*N* = 54	Hemicontusion	C7	KD	4:1 fat:carbohydrates + protein	70 g/kg body weight/d for 4 wk	Activated Nrf2 and suppressed NF-κB pathway, attenuating oxidative stress and inflammation; improved functional recovery.
Mayr et al., 2020 [68]	C57BL/6 mice (male, 8–16 wk)	*N* = 26	Hemisection	T10–T11	KD	6:1 fat:carbohydrates +protein	7 d before and 28 d after injury	No beneficial effects of KD on sensorimotor recovery in thoracic spinal cord hemisection or spared nerve injury.
Streijger et al., 2013 [69]	SD rats (male, ∼350 g)	*N* = 50	Hemicontusion	C5	KD	3:1 fat:carbohydrates + protein (#F5848; paste; Bio-Serv)	Started 4 h post injury for 14 wk	Improved forelimb motor recovery; reduced lesion area; increased grey matter sparing, GLUT1-positive blood vessels, and MCT1 expression; Pharmacological inhibition of MCTs blocked KD-induced neuroprotection.
Streijger et al., 2014 [70]	SD rats (male, ∼300 g)	*N* = 22	Contusion	C5	KD combined with ghrelin, ibuprofen, and C16	3:1 fat:carbohydrates + protein (#F5848; paste; Bio-Serv)	Started 4 h post injury for 10 wk	No significant improvements in histologic or functional outcome; Possible interactions among treatments.
Wang et al., 2017 [71]	SD rats (male, 8 wk, 290–310 g)	*N* = 72	Contusion	C5	KD, EODF, every-other-day ketogenic diet (EODKD) compared with Standard diet (SD)	KD: 4:1 fat:carbohydrates + protein	2 wk (KD, SD: ad libitum; EODF, EODKD: 24-h fasting, 24 h ad libitum)	KD, EODF, and EODKD increased βOHB levels; inhibited HDAC activity; increased histone acetylation; reduced oxidative stress; increased FOXO3a and Mt2 expression; reduced lipid peroxidation factors malondialdehyde (MDA) levels; promoted neuroprotection.
Yarar-Fisher et al., 2018 [72]	Human (AIS A-D, acute)	*N* = 7	Complete/incomplete	C4-T11	KD (70%–80% energy from fat)	Enteral feeding: ≈72% total energy as fat, ≈25% as protein, and ≈3% as carbohydrate solid feeding: ≈65% total energy as fat, ≈27% as protein, and ≈8% as carbohydrate and fiber	5 ± 2 wk	KD improved upper extremity motor scores, reduced inflammatory markers (↓fibrinogen, ↓CRP); promoted neuroprotection and functional recovery.
Kong et al., 2017 [73]	SD rats (male, 300–350 g)	*N* = 32	Hemicontusion	C5	KD	7:1 fat:carbohydrates + protein (Trophic Animal Feed High-Tech Co., Ltd.)	From 2 wk pre-SCI to 1 wk after SCI	Reduced oxidative stress markers and ROS products; suppressed Nox2 and Nox4 via HDAC inhibition; upregulated FOXO3a, MnSOD and catalase.
Seira et al., 2021 [74]	SD rats (male, ∼300 g)	*N* = 66	Contusion	C4-C6	KD	3:1 fat:carbohydrates + protein (F5848, Bio-Serv)	7 d post injury	Increased mitochondrial respiratory capacity; Increased mitochondrial biogenesis; Regulated mitochondrial-related genes whereas reducing oxidative stress.
Fisher et al., 2018 [75]	Human (chronic, long-standing SCI, AIS A/B)	*N* = 11	Complete	C5-T12	Isocaloric LC/HPD compared with combination exercise regimen	4:3:3 ratio by kcal from carbohydrates:protein:fat (carbohydrate-to-protein ratio <1.5)	1.6 g/kg protein per day for 8 wk	Comb-Ex increased type Ia myofiber distribution and CSA in VL muscle and type I and IIa myofiber CSA in deltoid muscle; Comb-Ex increased lean thigh mass, Vo2peak, and upper body strength; consuming HP diet alone had no effect on any of the outcome measures.
Li et al., 2018 [76]	Human (chronic, long-standing SCI, AIS A/B)	*N* = 11	Complete	C5-T12	LC/HPD compared with combined exercise regimen	4:3:3 ratio by kcal from carbohydrates:protein:fat (carbohydrate-to-protein ratio <1.5)	1.6 g/kg protein per day for 8 wk	HP diet improved insulin sensitivity; decreased TNF-α levels in patients with chronic SCI y. Comb-Ex decreased fasting glucose levels; no changes in GLUT4 translocation proteins.
Li et al., 2022 [77]	Human (chronic, AIS A-D, insulin-resistant)	*N* = 19	Complete/incomplete	C2-L2	LC/HPD	4:3:3 ratio by kcal from carbohydrates:protein:fat (carbohydrate-to-protein ratio <1.5)	8 wk	Altered gut microbiota: (increased *Bacteroides thetaiotaomicron, Coprococcus 3, Fusicatenibacter, Tannerellaceae*; decreased *Tyzzerella, Phascolarctobacterium, Romboutsia* ); improved insulin sensitivity in patients with chronic SCI.
Li et al., 2022 [78]	Human (chronic, AIS A-D, insulin-resistant)	*N* = 25	Complete/incomplete	C5-L2	LC/HPD	4:3:3 ratio by kcal from carbohydrates:protein:fat (carbohydrate-to-protein ratio <1.5)	8 wk (first 2 wk: 6-d-rotating menu; Beginning week 3: personalized LC/HP diet menu)	Reduced body fat and LDL cholesterol; no conclusion of effects on glucose metabolism and inflammation.
Smith et al., 2022 [79]	Long Evans rats (male, 250–300 g)	*N* = 36	Contusion	T10	LFD and HFD	HFD: 4.5:1.5:4 ratio by kcal from carbohydrates:protein:fat (#D03082706, Research Diets, 4.54 kcal/g) LFD: 7.6:1.5:0.9 ratio by kcal from carbohydrates:protein:fat (#D03082705, Research Diets, 3.81 kcal/g)	4-wk SCI recovery period, then HFD or LFD for 12 wk	Significant microbial changes at every taxonomic level; reduced gut microbiota diversity, worsened inflammatory response, exacerbated immune depression.
Dos Santos et al., 2022 [80]	Long Evans rats (male, 250–300 g)	*N* = 66	Contusion	T10	LFD or HFD for 12 wk	HFD: 4.5:1.5:4 ratio by kcal from carbohydrates:protein:fat (#D03082706, Research Diets, 4.54 kcal/g) LFD: 7.6:1.5:0.9 ratio by kcal from carbohydrates:protein:fat (#D03082705, Research Diets, 3.81 kcal/g)	4-wk SCI recovery period, then HFD or LFD for 12 wk	HFD: worsened metabolic dysfunction, reduced fat mass, affected cholesterol biosynthesis gene expression.
Graham et al., 2020 [40]	C57BL/6 mice (male, 3 mo)	*N* = 36	Transection	T10	HFD	HFD: 2:2:6 ratio by kcal from carbohydrates:protein:fat (#D12492, Research Diets, 5.21 kcal/g)	12 wk	Impaired glucose tolerance; reduced muscle and liver mass; no significant fat mass change compared with control diet.
Guo et al., 2019 [81]	SD rats (male, 4 wk, ∼70 g)	*N* = 200	Spared nerve injury	–	HFD vs. LFD	HFD: 45% kcal from fat (Research Diets, Inc) LFD: 10% kcal of fat (Research Diets, Inc)	12 wk	Enhanced neuropathic pain sensitivity via AMPK-CGRP pathway downregulation in spinal cord and dorsal root ganglion.
Harris et al., 2019 [82]	Long Evans rats (male, 12 wk, 250–300 g)	*N* = 40	Contusion	T10	HFD vs. LFD	HFD: 4.5:1.5:4 ratio by kcal from carbohydrates:protein:fat (#D03082706, Research Diets, 4.54 kcal/g) LFD: 7.6:1.5:0.9 ratio by kcal from carbohydrates:protein:fat (#D03082705, Research Diets, 3.81 kcal/g)	4-wk SCI recovery period, then HFD or LFD for 12 wk	LFD-SCI: reduced feed conversion efficiency; reduced muscle triglycerides and cholesterol. HFD: increased body fat percentage; exacerbated metabolic dysfunction; reduced lean mass; elevated fat mass. SCI rats preferred protein over Sham rats.
Person et al., 2020 [83]	Long Evans rats (male, 14 wk, 300–350 g)	*N* = 32	Contusion	T10	HFD	HFD: 4.5:1.5:4 ratio by kcal from carbohydrates:protein:fat (#D03082706, Research Diets, 4.54 kcal/g)	16 wk post injury	Worsened immune system function; reduced percentages of T cells, NK cells, and increased neutrophils; altered spleen and thymus gene expression.
Harris et al., 2023 [84]	Long Evans rats (male, 250–300 g)	*N* = 40	Contusion	T10	HFD	HFD: 4.5:1.5:4 ratio by kcal from carbohydrates:protein:fat (#D03082706, Research Diets, 4.54 kcal/g)	16 wk post injury	Worsened glycemic control; elevated blood glucose during GTT; altered glucagon and GLP-1 signaling in SCI rats.
Liu et al., 2021 [85]	C57BL/6 mice (male, 3 mo)	*N* = 48	Contusion	T9	HFD	HFD: 2:2:6 ratio by kcal from carbohydrates:protein:fat (#D12492, Research Diets, 5.21 kcal/g)	12 wk post injury	HFD exacerbated metabolic dysfunction, reduced FGF21 levels, impaired FGF21 signaling in liver and adipose tissue; reduced serum adiponectin and leptin levels; suppressed hepatic AdipoR2 mRNA expression and PPARa activation in the liver.
Spann et al., 2017 [86]	Long Evans rats (male, 400 g)	*N* = 32	Contusion	T10	HFD	HFD: 4.5:1.5:4 ratio by kcal from carbohydrates:protein:fat (#D03082706, Research Diets, 4.54 kcal/g)	4-wk SCI recovery period, then HFD for 8 wk	Exacerbated immune depression; reduced T-cell and NK cell activity; increased cortisol levels and cholesterol biosynthesis and immune cell trafficking pathways; changed gene expression in MMP12, APOC4, GPNMB, and IGF1 and 2
Ha Neui Kim et al., 2020 [87]	C57BL6J mice (10 wk, male and female)	*N* = 30	Lateral compression	T8-T9	HFHS diet	HFHS: 2:2:6 ratio by kcal from carbohydrates:protein:fat; contained 60% cholesterol and 19% sucrose (#D15010302, Research Diets)	From 7 wk pre-SCI to 4 wk after SCI	Impaired insulin signaling, TCA cycle function, and bladder recovery; increased microgliosis and oligodendrocyte loss; exacerbated myelin loss.
Totsch et al., 2017 [88]	SD rats (150–200 g, male and female)	*N* = 35	Inflammatory injury (complete Freund’s adjuvant injection)	–	SAD	SAD: 49:15.4:35.6 % kcal from carbohydrates:protein:fat (TD.140536, Envigo, Madison)	From 20 wk pretreatment to 4 wk post treatment	Increased fat mass, leptin levels, and proinflammatory cytokines; prolonged recovery from inflammatory injury.
Ni et al., 2023 [89]	C57BL/6 mice (female, 8–10 wk, 20–24 g)	*N* = 120	Contusion	T10	Resveratrol (MD)	–	200 mg/kg/d for 3 d post injury, i.p.	Inhibits ferroptosis and improves motor function by activating Nrf2/GPX4 pathway.
Zhang et al., 2019 [90]	SD rats (280–300 g)	*N* = 40	Contusion	T8	Resveratrol (MD)	–	200 mg/kg, 3 times per day, for 3 d post injury, i.p.	Alleviates LPS-induced inflammation, improves motor recovery, and inhibits IL-1β, IL-6, TNF-α via miR-132.
Kan et al., 2023 [91]	Wistar rats (female, 200–240 g)	*N* = 44	Contusion	T10	Resveratrol (MD)	–	10 mg/kg/d for 7 d post injury, i.p.	Reduces inflammation and apoptosis by inhibiting JNK/p38MAPK signaling pathway; improves motor recovery.
Yuan et al., 2021 [92]	SD rats (male, 200–220 g)	*N* = 105	Contusion	T10	IF	EODF: 24-h fasting intervals	From 8 wk preinjury to 4 wk after injury	Improved motor function recovery; reduced neuronal apoptosis; upregulated autophagy markers (LC3-II, Atg7); enhanced lysosome function; Activated AMPK/mTOR pathway and promoted TFEB expression.
Jeong et al., 2011 [93]	SD rats (male, 200–220 g)	*N* = 49	Contusion	T10	IF	EODF: 24-h fasting intervals Pair-fed CR: 75% of food intake	Post-EODF: for 10 wk post injury Pre-EODF: from 3 wk preinjury to 10 wk after injury Pair-fed: from 3 wk preinjury to 10 wk after injury	Improved functional recovery in hindlimb locomotion; increased serotonergic innervation, enhanced gray matter sparing without significant differences in lesion size. Promoted motor recovery via neuroplasticity and modulation of inflammation.
Streijger et al., 2011 [94]	C57BL/6 mice (male, 20–23g)	*N* = 29	Contusion	T10	IF	EODF: 24-h fasting intervals	For 15 wk post injury	Did not improve hindlimb motor recovery or increase ketone bodies after spinal cord injury, differing from effects seen in rat models. No significant benefits observed for neuroprotection or functional recovery.
Wang et al., 2023 [95]	SD rats (male, 2 mo, 130 g)	*N* = 72	Clamp	C5	IF	EODF: 24-h fasting intervals	For 12 wk post injury	Altered gut microbiota composition and enriched neuroprotective genes related to inflammation, neural growth, and apoptosis, promoting motor recovery post-SCI through modulation of gut–brain axis and transcriptome dynamics.
Zahner et al., 2020 [96]	SD rats (female, 100–250 g)	*N* = 60	Hemisection	T8	IF (EODF)	EODF: 24-h fasting intervals	For 1 wk or 7 wk post injury	Did not improve recovery of baroreflex regulation or sympathetic nerve activity, though it showed minor improvements in motor function in the acute phase post-SCI.
Plunet et al., 2010 [97]	SD rats (male, ∼300 g)	*N* = 46	Hemisection	C4	IF (EODF)	EODF: 24-h fasting intervals	From 4 wk preinjury to 6 wk after injury	Improved motor recovery, Promoted corticospinal tract plasticity, Increased β-hydroxybutyrate levels, reduced lesion volume by 50%. Enhanced neuroprotection and plasticity were observed.
Zhang et al., 2023 [98]	C57BL/6 mice (female, 6–8 wk, 20–25 g)	*N* = 276	Contusion	T9–T10	CR	CR mimetics: 3,4-dimethoxychalcone	200 mg/kg, from half an hour before surgery to 3 d post injury	Promoted functional recovery by upregulating TFEB-mediated autophagy and inhibiting pyroptosis and necroptosis after SCI.
Liu et al., 2017 [99]	SD rats (male, 240-260 g)	*N* = 16	Chronic constriction injury of sciatic nerve	–	CR	60% intake	From 6 wk preinjury to 120 h after injury	Reduced mechanical and thermal hypersensitivity. Increased SIRT1 expression and reduced neuroinflammation, mitochondrial ROS, and IL-1β levels, leading to decreased pain sensation.

Table 1 shows summary of studies involving dietary patterns and their effects on gut microbiota and spinal cord injury (SCI) recovery. “SCI Model Method” refers to the type of SCI induced, such as hemicontusion or transection. “SCI Damaged Segment” indicates the specific level of spinal injury.

Abbreviations: AIS, American Spinal Injury Association (ASIA) Impairment Scale; AMPK, AMP-activated protein kinase; CGRP, calcitonin gene-related peptide; CR, caloric restriction; CRP, C-reactive protein; CSA, cross-sectional area; EODF, every-other-day fasting; EODKD, every-other-day ketogenic diet; GLP-1, glucagon-like peptide-1; GTT, glucose tolerance test; HPD, high-protein diet; HFD, high-fat diet; HFHS, high-fat high-sugar diet; IF, intermittent fasting; KD, ketogenic diet; KS, ketone salt; LC/HP, low-carbohydrate/high-protein diet; LFD, low-fat diet; MCT, monocarboxylate transporter; MD, Mediterranean diet; mTOR, mechanistic target of rapamycin; NK, natural killer (cells); ROS, reactive oxygen species; SD, Sprague–Dawley rats; TCA, tricarboxylic acid cycle; TFEB, transcription factor EB; VL, vastus lateralis (muscle).

#### Effects of Mediterranean diet on SCI

The Mediterranean diet (MD) emphasizes plant-based foods (fruits, vegetables, legumes, nuts), olive oil, and fish, whereas limiting red meat, dairy, and saturated fats. Known for its health benefits, this pattern is often used to manage chronic diseases and reduce inflammation [100]. Plant foods and extra virgin olive oil (EVOO) provide vitamins and antioxidants that boost antioxidant enzyme expression and reduce inflammation through the Nrf2 pathway [101,102]. EVOO also enhances the absorption of carotenoids and other bioactive compounds, supporting redox balance in individuals with SCI [103].

Resveratrol, found in grapes, nuts, dried fruits, and red wine, is another key component. Animal studies show that it reduces oxidative stress, inhibits cell damage, and lowers inflammatory mediators by influencing pathways such as Nrf2/HO-1, NRF2/GPX4, JNK/p38MAPK, and JAK2/STAT3 [91,89].

The high-fiber content in the MD supports digestive health, which is crucial for patients with SCI who often struggle with neurogenic bowel dysfunction. However, some may experience discomfort from increased fiber intake, explaining why plant consumption may be low in this group [4,6]. Additionally, certain herbs and specific fruits (such as grapefruit) contain active components (e.g. glucosinolates, flavonoids, and furanocoumarins) that can affect drug metabolism and bioavailability [104], and therefore should be considered in dietary planning.

The MD helps reduce various inflammatory markers [V‌ascular ‌E‌ndothelial ‌G‌rowth ‌F‌actor (‌VEGF), C-reactive protein (CRP), M‌onocyte ‌C‌hemoattractant ‌P‌rotein-1 (‌MCP-1), IL-2, IL-17, IP-10, and IL-12] and is associated with higher levels of the anti-inflammatory cytokine IL-10 in clinical studies [105,106]. Combining this diet with exercise and supplementation can be especially effective in patients with SCI, improving muscle strength, flexibility, and cardiometabolic health [6].

In summary, the MD’s antioxidant- and anti-inflammatory-rich components—such as olive oil, resveratrol, and high-fiber plant foods—can reduce oxidative stress and inflammation in SCI. However, as its specific effects on individuals with chronic SCI remain unexplored, its role in neurorepair and recovery remains incompletely understood.

#### Effects of a high-protein diet on SCI

A high-protein diet (HPD) increases daily protein intake to 18%–35% or more of total energy [107], often pairing high-quality protein sources with reduced carbohydrate consumption [77]. This approach helps maintain muscle mass, enhances metabolic function, and is commonly used for fat loss and enhanced physical training [107]. However, studies suggest that increasing protein intake without adjusting other macronutrients may elevate the risk of neurogenic obesity [108]. The following section will explore the benefits and drawbacks of HPD.

Clinical studies on patients with chronic SCI suggest that HPDs enhance insulin sensitivity, lower TNF-α (an inflammation marker), and reduce visceral and total body fat [76]. HPDs also help alter body composition and reduce LDL cholesterol levels [109]. In chronic SCI, combining HPD with exercise can further promote muscle growth and fiber-type changes in paralyzed muscles, whereas also lowering fasting blood glucose more effectively than diet or exercise alone [75,76]. There are currently no relevant studies on the acute phase, highlighting the need for further research.

Despite these advantages, HPD presents challenges for patients with SCI, especially those with comorbid conditions like neurogenic bladder, chronic urinary tract infection, and kidney stones, because excessive protein intake can increase glomerular pressure and hyperfiltration, potentially exacerbating chronic kidney disease over time [110]. Patients with SCI also face pressure injuries, requiring careful protein adjustments to support wound healing whereas aligning with treatment goals. [111]. Unbalanced increases in dietary protein without reducing other macronutrients may lead to excessive caloric consumption and elevate the risk of neurogenic obesity [108]. Even under isocaloric conditions, HPDs may not prevent muscle wasting associated with long-term paralysis in SCI [75]. Furthermore, the timing of HFP intervention is crucial. In the acute phase of injury, patients often experience obligatory negative nitrogen balance, leading to muscle loss, where increased protein intake may fail to counteract catabolism and instead result in overfeeding [108,112]. Moreover, higher intake of certain amino acids (e.g. tryptophan, isoleucine, lysine, tyrosine, threonine, leucine, methionine, and valine) is linked to reduced bone mineral density in the lumbar spine in patients with SCI [113]. In contrast, the hip and femur regions appear unaffected. Long-term effects on glucose metabolism and inflammation also remain unclear.

In essence, although HPD can enhance metabolic markers, body composition, and muscle mass in patients with SCI, careful attention must be paid to total intake, its proportion of total energy supply, timing, complementary exercise, and protein source (e.g. plant compared with animal and their amino acid profiles), as different sources may differentially impact the gut microbiome (discussed further in the Effects of dietary patterns on gut microbiota section). Clinicians should also monitor bone health, renal function, and the potential risk of neurogenic obesity to ensure that long-term metabolic outcomes remain favorable.

#### Effects of ketogenic diet on SCI

The ketogenic diet (KD) is a high-fat (80%–90%), low-carbohydrate, moderate-protein eating pattern that increases ketone body production (e.g. β-hydroxybutyric acid, βOHB) and enhances mitochondrial oxidative metabolism [114]. Originally used to treat refractory epilepsy, the KD has shown neuroprotective potential in conditions like Alzheimer’s disease, Parkinson’s disease, amyotrophic lateral sclerosis, and SCI [62,69,[115], [116], [117], [118], [119]]. Research indicates that KD can enhance motor function, reduce pain and neuroinflammation, and lower both functional impairment and mortality rates in patients with SCI [117].

Evidence from both acute and chronic SCI models supports KD’s neuroprotective role. In a rat model, KD reduced oxidative stress and inflammation within 3 d of injury, increased forelimb mobility, and maintained functional gains for ≤12 wk after returning to a normal diet [65,69]. Histological studies reveal that KD can lessen spinal cord lesions, protect gray matter cells, and promote glial cell survival, particularly in cervical spine injuries of rats [65,120]. Nonetheless, findings in mouse models have been mixed. In a lower limb nerve injury model of mice, KD did not significantly enhance sensory or motor outcomes, possibly due to differences in injury type, intervention duration, or effects on neurotransmitters [68]

Animal studies indicate KD’s neuroprotective mechanisms are closely linked to mitochondrial function [67,74,121]. Within 7 d of SCI, KD restored mitochondrial respiratory capacity, increased mitochondrial biosynthesis, and lowered oxidative stress by activating energy metabolism genes (e.g. HCA2, VDAC1), stimulating electron transport chain complexes, and triggering the NRF2-dependent antioxidant pathway. These changes help reduce reactive oxygen species levels [74]. In addition, KD’s primary ketone body, βOHB, inhibits class I histone deacetylases, influencing the expression of genes like FOXO3a, NOX2, and NOX4 and further strengthening antioxidant defenses [73].

KD also regulates inflammatory responses in animal studies. By activating the Nrf2 pathway, it dampens oxidative stress and secondary inflammation after nerve injury [67,74]. KD has been shown to inhibit Nuclear Factor-κappa B (NF-κB) signaling, lowering TNF-α, IL-1β, and Interferon-γamma (IFN-γ) levels [67]. βOHB also prevents NLRP3 inflammasome activation and encourages macrophage/microglia polarization toward a protective M2a subtype, thus easing neuroinflammation in SCI rats models [65,120].

Metabolomics research highlights KD’s profound effects on energy metabolism and nutrient integration. KD can modify the immune environment and support myelin regeneration in SCI rats by modulating steroid synthesis and reducing expression of immune-related pathways [65]. Animal studies suggest enhanced fatty acid oxidation in mitochondria and peroxisomes increases ketone body production, allowing neurons to bypass glycolysis and tap directly into the tricarboxylic acid (TCA) cycle for efficient energy supply [114,122]. However, KD may influence bone health. One study found that KD delayed osseointegration after lumbar spinal fusion in rats and reduced bone mass at 4 wk postsurgery, without changing serum calcium or phosphorus levels [66].

A short-term (12.9 d) KD intervention in the acute phase increased American Spinal Injury Association scores and raised blood ketone levels without major metabolic disturbances, though some patients experienced gastrointestinal issues, hypoglycemia, or urticaria [123]. A longer-term clinical study (5 ± 2 wk), conducted during the acute phase, confirmed KD’s safety and noted improvements in upper limb motor function and anti-inflammatory markers [72]. Clinical research on KD in chronic SCI is currently lacking.

In summary, KD shows promise in enhancing mitochondrial function, antioxidant defenses, and inflammatory regulation in SCI. However, its efficacy can vary by injury model, and the long-term safety remains uncertain. Future studies should refine intervention strategies, investigate epigenetic mechanisms, and determine the feasibility of sustained KD use in clinical practice.

#### Effects of intermittent fasting and caloric restriction on SCI

Dietary restriction strategies, such as intermittent fasting (IF) and caloric restriction (CR), have gained attention for their ability to extend lifespan and lower the risk of chronic conditions like cardiovascular disease and cancer [124,125]. These interventions have also shown benefits in models of neurotraumatic diseases, such as traumatic brain injury [126]. In addition, dietary restriction strategies have shown promise in addressing neuroprotection, inflammation control, and gut microbiota regulation in SCI [93,97].

IF is generally easier to maintain than prolonged fasting because it primarily extends the interval between meals without causing significant gastrointestinal harm [127,128]. In rat models, every-other-day fasting (EODF) reduced SCI lesion areas, encouraged corticospinal tract sprouting, and increased sensory and motor performance [93,97,129]. In the acute phase, EODF also decreased caspase-dependent apoptosis and receptor-interacting protein kinase-dependent necroptosis, lowered the expression of apoptotic markers (e.g. cleaved caspase-3, Bax/Bcl-2 ratio), and enhanced autophagy via increased LC3-II and Beclin-1 [92,130]. These improvements are partly attributed to enhanced lysosomal activity driven by AMPK/mTOR pathway activation and T‌ranscription ‌F‌actor ‌EB‌ (‌TFEB) upregulation [92,130]. However, in mice, EODF did not yield similar benefits, possibly due to species-specific metabolic responses and the lack of significant ketone body elevation during fasting [94,96].

CR, which involves strictly reducing total daily energy intake, can induce more pronounced weight loss than IF but is harder to sustain over time and less suitable for chronic conditions [128]. Nevertheless, CR has been shown to alleviate post-incisional pain in rats after plantar incision and to enhance the efficacy of analgesics [99]. In animal models, CR appears to reduce neuroinflammation and neuropathic pain in rats through mechanisms involving increased SIRT1 expression, decreased mitochondrial reactive oxygen species production, inhibited NF-κB activation, and reduced IL-1β levels in the spinal cord, whereas also modulating spinal glial cell activity [131]. In a recent study, CR mimetics have been shown to reduce glial scar area and motor neuron death, promote autophagy to inhibit pyroptosis and necroptosis, and improve functional recovery in mice after SCI [98].

In summary, IF and CR offer potential benefits for SCI rehabilitation by influencing metabolism, inflammation, and autophagy. Although their mechanisms and outcomes differ, the lack of clinical and animal research on SCI in both the acute and chronic phases warrants further investigation to determine optimal protocols and clarify their roles in clinical management.

#### Effects of high-fat diet and high-fat, high-sugar diet on SCI

A high-fat diet (HFD), in which 30%–60% or more of total energy comes from fat, can influence neurological health and worsen dysfunction after SCI [132]. In a rat model, animals fed a diet with 40% fat developed increased body fat over 12 wk compared with those on a low-fat diet (8% fat), suggesting that high-fat intake may promote metabolic disturbances [82]. Although locomotor activity was lower in the HFD-SCI group, their overall energy expenditure remained similar to that of the sham-HFD group, indicating that HFD might alter energy use in ways unrelated to exercise. After 15 wk, SCI rats on the HFD showed elevated insulin, C-peptide, and leptin levels, all associated with metabolic syndrome. Impaired glucose tolerance, along with higher levels of insulin-like growth factors (IGF-1 and IGF-2) at the injury site, further reflects these metabolic imbalances [40,86].

HFDs also appear to disrupt cholesterol metabolism. For instance, SCI rats fed HFD showed increased expression of ABCA1 and APOC4 and higher levels of lecithin-cholesterol acyltransferase, whereas key enzymes for cholesterol synthesis (HMGCS1 and HMGCR) were downregulated [86]. This imbalance may worsen neurological damage after SCI.

Additionally, high-fat intake has been linked to poor myelin repair and abnormal immune responses. One study found that an HFD disrupted astrocyte and oligodendrocyte function via a NAD+-dependent CD38 mechanism, leading to myelin defects and impaired nerve repair in rats with SCI [133]. HFD also heightened immune cell activation and infiltration in SCI rats, altered gene expression in immune organs, and caused thymus degeneration and enlarged spleens [83]. In these rats, increased expression of proinflammatory genes (e.g. TLR4, CD14), changes in macrophage markers (e.g. CD 68), and shifts in matrix metalloproteinases suggested impaired immune surveillance and tissue remodeling, delaying the recovery process [83].

Not all studies have found a clear link between HFD and inflammation. A short-term HFD study in patients with chronic SCI did not show significant changes in inflammatory markers, even though metabolic markers worsened after meals. In this case, abnormal blood glucose responses after eating may have influenced high-sensitivity CRP levels more than the diet itself [134]. This suggests that although HFDs often contribute to metabolic dysregulation, their direct impact on inflammation may vary.

The high-fat, high-sugar (HFHS) diet includes large amounts of fat, sugar, and salt, along with refined carbohydrates and low fiber, vitamins, and minerals. This pattern is closely associated with chronic diseases like heart disease, type 2 diabetes, and obesity [135]. Animal studies indicate that HFHS diets produce metabolic instability (e.g. fluctuating blood glucose and lipids), impaired pancreatic function, reduced glucose tolerance, and insulin resistance [87,88]. In SCI mice models, HFHS diets worsen oligodendrocyte and myelin loss, slow axonal repair, and hinder sensory, motor, and bladder function recovery [87]. HFHS diets may also trigger persistent activation of central and peripheral immune systems, increasing susceptibility to chronic pain in rats with SCI [88].

In summary, both high-fat and HFHS diets can negatively affect metabolism, nerve repair, and immune function in SCI. Although they may provide short-term energy benefits, their long-term consequences are largely harmful, and related clinical research remains scarce. In the future, establishing a large-scale public database of patients with SCI may facilitate dietary screening and matching to better assess the clinical impact of these diets. Reducing intake of high-fat, processed foods and increasing healthier fats and dietary fiber can be a key strategy for improving recovery and overall health in individuals with SCI.

#### Effects of other dietary patterns on SCI

Beyond the previously discussed dietary approaches, several other patterns—including anti-inflammatory diets, plant-based diets, the Dietary Approaches to Stop Hypertension (DASH) diet, and low-carbohydrate diets—offer distinct nutritional profiles that may influence SCI recovery [[136], [137], [138], [139]].

Anti-inflammatory diets emphasize fruits, vegetables, whole grains, and healthy fats whereas limiting processed foods, sugars, and red meat [140]. They often incorporate foods and supplements known to reduce inflammation, such as omega-3 fatty acids, coenzyme Q10, and N-acetylcysteine [139]. Research shows that a 12-wk anti-inflammatory dietary intervention significantly eased neuropathic pain in patients with SCI, possibly by lowering proinflammatory cytokines (e.g. IFN-γ, IL-1β, and IL-6) and prostaglandin E2 [141]. Another randomized clinical trial found that this approach lowered fat intake, increased protein intake, and reduced markers of chronic inflammation (e.g. IFN-γ, IL-1β) in patients with SCI [103]. Moreover, many elements of anti-inflammatory diets can improve gut health in patients by modulating the microbiota and enhancing short-chain fatty acid (SCFA) production [102,[142], [143], [144]].

Currently, there is a lack of data on how other patterns—such as plant-based diets, DASH diets, and other emerging dietary approaches—affect SCI outcomes. Their unique benefits and potential risks in the context of SCI are still unclear and warrant further exploration.

In conclusion, different dietary patterns can significantly influence recovery and long-term outcomes in SCI. The MD, rich in antioxidants and anti-inflammatory compounds, shows promise by enhancing redox balance and reducing inflammation. HPDs may enhance insulin sensitivity and body composition, especially when combined with exercise. KDs appear to offer neuroprotective benefits by reducing oxidative stress and inflammation, though their precise mechanisms and long-term safety remain to be fully understood. Finally, other diets—such as anti-inflammatory, plant-based, and DASH—also hold potential but require more rigorous investigation. Tailoring dietary strategies to each individual remains critical for optimizing SCI rehabilitation and overall well-being.

## Effect of Diet on Gut Microbiota after SCI

In the previous section, we explored the diverse impacts of diet on SCI recovery. Although dietary components can exert direct effects on SCI pathophysiology, they may also shape the gut microbiota—whose metabolites and interactions with the intestinal environment can profoundly influence outcomes. Understanding the changes in the intestinal microbiota after SCI is essential before examining how dietary modifications can improve it.

SCI alters patients’ metabolic needs and eating patterns, although also disrupting the gut microbial balance. This dysbiosis not only affects nutrient absorption and metabolism [[25], [26], [27]] but also worsens neurogenic bowel dysfunction via the “microbiota-gut-brain axis.” As a result, appetite, digestion, and nutrient uptake suffer, creating a cycle in which dysbiosis and nutritional problems reinforce each other [3,19,145,146].

Adjusting dietary patterns can help restore healthier gut microbiota. Different diets, through various nutrients and their interactions, may support SCI recovery by improving microbial composition and function [59,102,147]. The following sections outline the changes in gut microbiota after SCI and the potential role of diet in correcting these imbalances.

### Changes and effects of gut microbiota after SCI

SCI profoundly alters patients’ metabolic demands and eating patterns while disrupting the balance of their gut microbiota. Numerous studies in both animal models and human have shown that SCI reduces both the abundance and diversity of gut microbiota, with an increase in the abundance of *Clostridia* [[148], [149], [150]] and a decrease in the abundance of *Proteobacteria* [[150], [151], [152]]. At the same time, bacteria associated with inflammation and potential pathogenicity—such as *Enterococcus, Streptococcus, Klebsiella, Streptococcaceae, Methanobacteriaceae, Enterobacteriaceae, and Verrucomicrobiaceae*—rise in prevalence. Conversely, populations of SCFA-producing bacteria like *Prevotellaceae* and *Ruminococcaceae* decrease [23].

SCI also affects the ratio of *Firmicutes* to *Bacteroidetes* (F/B), a commonly used indicator of gut health [153]. Imbalances in this ratio compromise the gut’s microbial ecosystem, weaken the intestinal barrier, and promote the release of proinflammatory cytokines [154,155].

These microbial shifts influence key metabolic products. In SCI models, LPS levels increase whereas SCFA levels decrease [25,156]. In combination with the activation of immune pathways (e.g. Toll-like receptors, NLRP3 inflammasomes) [[157], [158], [159]], these changes lead to autonomic dysfunction and heightened intestinal permeability, thereby fueling neuroinflammation [154,160].

Gut dysbiosis in patients with SCI is closely tied to secondary injury mechanisms, including neuroinflammation and compromised intestinal barrier function, and thus affects overall patient outcomes [20,148,161]. Disruptions in the “microbiota-gut-brain axis” can contribute to metabolic disorders, heightened infection risk, and further neurological damage [20]. Indeed, the degree of dysbiosis often correlates with the severity of neurological impairment in patients with SCI [23].

This imbalance in gut microbiota is driven not only by the SCI itself—through immune disruptions, neurogenic bowel dysfunction, and metabolic disturbances—but also by postinjury nutritional issues. For example, patients often have inadequate nutrient intake, leading to deficiencies such as low vitamin D levels, which may reduce Lactobacillus abundance [42]. A decline in Lactobacillus can, in turn, lower the synthesis of B vitamins and vitamin K, worsening the patient’s overall health [147,162].

In sum, gut dysbiosis is both a consequence and a contributing factor in SCI progression, influencing nutrient metabolism, immune regulation, and the integrity of the intestinal barrier [20,23,149]. However, understanding these relationships remains challenging due to a lack of long-term human studies and the inherent complexity of SCI. Larger-scale research is needed to better characterize gut microbiota changes in diverse SCI populations and guide future dietary interventions aimed at restoring microbial balance and improving patient outcomes.

### Overview of dietary influences on gut microbiota

Diet plays a critical role in shaping the gut microbiota’s composition and function. Different nutrients exert distinct effects on microbial populations, and when combined into specific dietary patterns, their influences can be synergistic or more complex than a simple sum of individual parts [19]. Exploring how various dietary patterns and individual nutrients specifically impact microbiome can guide the development of targeted nutritional interventions.

#### Effects of dietary patterns on gut microbiota

Research indicates that various dietary patterns can significantly alter the gut microbiome’s composition, influencing inflammation, metabolism, and overall gut health [19]. Although direct evidence in individuals with SCI is limited, understanding these general patterns can help guide targeted dietary interventions for patients with SCI.

##### Effects of MD on gut microbiota

Several clinical studies have shown that stronger adherence to the MD is associated with increased abundance and overall bacterial counts in the gut microbiota [[163], [164], [165]]. In particular, the MD can boost populations of *Bifidobacterium, Faecalibacterium prausnitzii, and Christensenellaceae* [163,164]. These bacteria support the production of SCFA, stimulate anti-inflammatory factor expression, and help maintain immune balance and gut health. At the same time, the MD reduces *E. coli, Clostridium spp., and Streptococcaceae*, as well as lowers the F/B ratio in nonhuman primates [165], which is associated with a healthier gut environment and reduced risk of inflammation-related diseases [155].

Additionally, higher adherence to the MD is usually accompanied by greater SCFA levels and relatively lower levels of branched-chain fatty acids and bile acids in feces [166]. Increased SCFA contribute to stronger anti-inflammatory effects and help preserve the integrity of the intestinal mucosal barrier [167], potentially mitigating inflammation secondary to SCI [25]. Specific MD components may influence particular microbial taxa; e.g. olive oil consumption correlates with certain Fusobacterium species, and red wine consumption correlates with *E. faecalis* [168]. Moreover, resveratrol has been shown to mitigate SCI-related gut dysbiosis and reduced butyrate levels, enhance the intestinal barrier, and support functional recovery in patients with SCI [169,170].

In addition to these components, the fiber-rich plant components within the MD can produce SCFA, such as acetate and butyrate, upon degradation. These SCFAs lower colonic pH and create a favorable environment for bacteria like *Bifidobacteria* and other SCFA-producing taxa [171]. However, as mentioned previously (Effects of MD on SCI section), high-fiber intake may exacerbate neurogenic intestinal symptoms in some patients with SCI [6]. This effect may be related to the physical properties of fiber—for instance, soluble fiber (e.g. oats, pulses) absorbs water and forms gels, increasing stool stickiness [172]. It may also be due to the production of gases such as carbon dioxide, hydrogen, and methane when fiber-degrading flora break down dietary fiber in the intestines [173]. Moreover, excessive fiber intake can slow gas transit, “trapping” gas and intensifying bloating [173]. Although increasing fluid intake alongside a high-fiber diet can help alleviate intestinal discomfort [174], patients with SCI often have neurogenic bladders, and excessive fluid intake is not conducive to managing this condition [175]. Therefore, a dietary strategy that gradually increases fiber content and avoids sudden large intakes is essential for patients with SCI.

These findings suggest that adjusting MD-related components may help regulate gut microbiota after SCI, although the precise mechanisms warrant further investigation.

##### Effects of HPD on gut microbiota

By contrast, the effect of a HPD on gut microbiota depends largely on the protein source. Animal studies indicate that diets high in animal proteins (HPD-a) markedly decrease intestinal bacterial diversity in mice whereas increasing the abundance of *Enterococcus, Streptococcus, Turicibacter, Escherichia coli, and Peptostreptococcaceae* [176]. The change of *Peptostreptococcaceae* is strongly linked to higher risks of intestinal inflammation and metabolic disturbances [177]. Clinical research supports these findings, showing that HPD-a leads to more bile-tolerant microbes (*Bacteroidetes, Alistipes, Bilophila*) and fewer fiber-fermenting bacteria (*Roseburia, Eubacterium rectale, Ruminococcus bromii*). This shift raises trimethylamine-N-oxide levels and lowers SCFA levels [[178], [179], [180]], suggesting that the overall impact of HPD-a is not favorable for gut homeostasis or metabolic health.

In contrast, high plant-based protein diets (HPD-p) increase the abundance of *Bifidobacteriaceae* and *Desulfovibrionaceae* in human gut, as well as *Lactobacillus spp., Lachnospiraceae,* and *Erysipelotrichaceae* [181,182]. These microbes support intestinal immunity and reduce inflammation. HPD-p also curbs opportunistic pathogenic species such as *Pseudomonas fragilis and Clostridium perfringens* [181,182], thereby lowering the risk of infections and inflammation. Furthermore, HPD-p enhances SCFA production in animal model by promoting bacteria that generate acetate (e.g. *Akkermansia, Bifidobacterium spp.*) [176] and butyrate (e.g. *Coprococcus*) [183]. One study found that SCFA levels in HPD-p-treated mice were much higher than in their HPD-a counterparts [176].

Notably, similar to the previously mentioned effects of high-fiber in the MD on gut microbiota, HPD-p also promotes the growth of *Bifidobacteria, Lactobacillus*, etc. [171,182]. Studies show that plant-based proteins consumed in daily meals are commonly accompanied by dietary fiber [184,185], and current research separating the specific effects of plant proteins from fiber remains limited. Therefore, it cannot be excluded that high-fiber components may lead to intestinal symptoms that do not occur in HPD-p and further studies on this distinction are warranted. In conclusion, although the HPD-p based strategy is more helpful than HPD-a in maintaining the balance of intestinal flora and reducing the risk of inflammation in patients with SCI, patients with SCI should introduce these diets gradually and ensure adequate fluid intake to minimize initial gastrointestinal discomfort.

##### Effects of HFD and HFHS diet on gut microbiota

HFD and HFHS diets display characteristic patterns of influence on the gut microbiota. Research shows that HFDs, in both humans and animal models, reduce overall bacterial diversity and lower *Bifidobacteria, Lactobacillus,* and *Lactococcus*. Meanwhile, they increase potentially harmful bacteria such as *Enterobacteriaceae, Clostridium*, and *Fusobacterium*, as well as elevate the F/B ratio [186,187]. These shifts can diminish anti-inflammatory microbes and compromise gut metabolic function, thereby negatively impacting systemic health.

Similarly, HFHS diets decrease microbial diversity and favor a Bacteroides immitis-dominated community, an effect attributed more to the diet itself than to obesity [188]. Clinical studies indicate that HFHS patterns raise levels of bile-tolerant bacteria (*Collinsella, Parabacteroides, Bilophila wadsworthia*) and reduce fiber-fermenting species (*Faecalibacterium prausnitzii, Lachnospiraceae, Butyricoccus*), further increasing the F/B ratio [[188], [189], [190]]. The high-fat and low-fiber, plant protein intake typical of HFHS can promote inflammation and harm both gut and metabolic health [191]. Moreover, the ultraprocessed foods characteristic of HFHS have been linked to greater abundance of Firmicutes (e.g. *Blautia, Coprococcus, Dialister, Megasphaera, Oscillospira, Roseburia, Faecalibacterium prausnitzii*), which correlates with lower levels of SCFA, indole-3-lactic acid, and indole-3-propionic acid in human [192,193]. These changes can adversely affect neurological function, cognition, and behavior [172,173], providing insight into the severe cognitive dysfunction often seen in patients with SCI [194].

Because patients with SCI often consume diets rich in simple carbohydrates and fats but low in fiber, and are prone to micronutrient deficiencies [16], their dietary patterns resemble HFHS characteristics. The gut dysbiosis observed in SCI, including reduced bacterial diversity, fewer SCFA producers, more opportunistic pathogens, and higher F/B ratio [23,148,151], aligns with the microbial changes induced by HFHS. Adjusting the dietary habits of patients with SCI toward increased fiber and plant-based foods may help restore microbial balance and support their recovery.

##### Effects of KD on gut microbiota

The KD also warrants attention. Unlike standard HFD or HFHS patterns, KD can significantly alter gut microbiota composition, affecting the abundance of *Actinobacteria, Bacteroidetes, and Firmicutes*, and changing fecal metabolite profiles in both human and mice [195]. Although *Bifidobacteria* generally promote gut health [196], some evidence suggests that ketone bodies can selectively inhibit certain *Bifidobacteria* strains associated with proinflammatory Th17 cell induction, thereby conferring anti-inflammatory benefits [195]. According to a review that includes both animal and human research, KD’s microbial changes—e.g. boosting *Akkermansia muciniphila* and lowering F/B ratio—have shown promise in managing epilepsy, Alzheimer’s, autism, obesity, and other conditions [197]. However, the low-fiber intake characteristic of the KD may lead to gut microbiota alterations similar to those observed with HFD or HFHS diets, such as decreased *Firmicutes* and increased *Proteobacteria* [191]. Nevertheless, the extent to which this factor contributes to KD-induced microbial changes remains unclear. Although KD’s benefits for SCI have been studied [117], the specific role of gut microbiota in mediating these effects has yet to be fully understood. Additionally, long-term KD use can risk intestinal dysbiosis and complications like bone health issues and kidney stones—concerns particularly relevant to patients with SCI [116].

In summary, although KD may offer short-term improvements in gut microbiota and SCI outcomes, it must be applied cautiously and monitored closely for adverse effects. Regular assessment of gut microbiota, bone mineral density, blood calcium, and related health indicators is critical to balancing KD’s potential benefits and risks in the clinical management of SCI.

##### Effects of IF and CR on gut microbiota

Studies have shown that dietary restriction interventions like IF and CR can positively influence gut microbiota composition and function without altering the actual composition of one’s diet. By modifying meal frequency and energy intake alone, both IF and CR can bring about notable changes in microbial communities.

Numerous studies in both animal models and human indicate that IF has been associated with increased alpha-diversity in the gut microbiota, promoting the growth of *Lactobacillus and Bifidobacterium,* lowering the F/B ratio, and reducing opportunistic pathogens [[198], [199], [200], [201]]. It also stimulates the production of metabolites like SCFA, indole-3-propionic acid, and 5-HT, all of which contribute to enhanced gut barrier integrity and immune modulation [202]. In rat models of SCI, EODF (a form of IF) increased anti-inflammatory strains like *Lactobacillus and Lachnospiraceae* whereas reducing proinflammatory species like *Bacteroides simulans*, thus creating a more favorable inflammatory environment [95].

CR, although having a milder effect on microbial diversity compared with IF, also enriches the gut with SCFA-producing and anti-inflammatory bacteria (e.g. *Akkermansia, Bifidobacterium, Roseburia, Lactobacillus, Bacteroides*) and reduces opportunistic pathogens [203]. This shift supports metabolic improvements, including enhanced fatty acid catabolism, gluconeogenesis, β-oxidation, and inhibition of lipid biosynthesis—all potentially beneficial to patients with SCI [203,204].

##### Effects of anti-inflammatory diets on gut microbiota

Additionally, components of anti-inflammatory diets, such as ω-3 PUFAs, can further bolster anti-inflammatory effects by favorably modulating the gut microbiota and increasing SCFA production [102,142,143,144], as will be discussed in detail in the following sections. However, the complexity of interactions between different anti-inflammatory nutrients and microbial populations means further research is needed. The combined, sometimes conflicting, and individually variable effects of these components on the gut microbiome must be better understood for safe and effective long-term application in patients with SCI.

In conclusion, various dietary patterns, including IF, CR, and anti-inflammatory diets, have the potential to ameliorate gut dysbiosis and improve metabolic homeostasis in individuals with SCI. Yet, the intricate interplay between the microbiota and the host under different dietary regimens remains an area in need of deeper investigation. Future large-scale, long-term studies will be essential for identifying the most effective and personalized dietary strategies to restore microbial balance and promote optimal recovery and health in patients with SCI.

#### Effects of specific nutrients on gut microbiota

Analyzing the roles of individual nutrients on gut microbiota, beyond the broader patterns discussed, can deepen our understanding of their synergistic or antagonistic effects within dietary patterns. This knowledge could guide more precise dietary interventions to optimize intestinal health, especially in the context of SCI.

##### ω-3 PUFA

Omega-3 PUFAs can significantly influence gut microbiota composition. Clinical studies report that ω-3 PUFAs supplementation increases the abundance of *Bacteroides, Bifidobacterium, and Roseburia*, whereas reducing *Faecalibacterium* [144]. In rat models, ω-3 PUFAs not only elevate levels of *Bifidobacteria and Akkermansia muciniphila*, but also enhance the intestinal environment by increasing mucosal thickness and modulating genes involved in fat metabolism [205,206]. Further evidence from patients with inflammatory bowel disease shows that ω-3 PUFAs help restore a healthier F/B ratio and improve the microbial community structure [207].

By enriching butyrate-producing bacteria—including *Blautia, Bacteroides, Roseburia,* and *Coprococcus*—ω-3 PUFAs boost the production of SCFA, which are vital for gut barrier integrity and anti-inflammatory effects in rats model [28]. Additionally, clinical studies show that ω-3 PUFAs can inhibit LPS-induced production of proinflammatory cytokines (e.g. TNF-α) and suppress the NF-κB and MAPK signaling pathways, thereby reducing inflammation, lowering intestinal permeability, and stabilizing the gut microbiota [208,209].

Given these findings, ω-3 PUFA supplementation holds potential for supporting nerve repair and improving the intestinal environment in patients with SCI. However, more research is needed to fully elucidate its specific mechanisms of action and to confirm its benefits in clinical practice.

##### Vitamins

Vitamins and the intestinal microbiota influence each other in both directions. On one hand, gut microbes can synthesize certain vitamins—such as vitamin K and B vitamins [147]—and affect vitamin absorption, metabolism, and function by regulating intestinal pH, gastric emptying, and other physiological processes [210,211]. On the other hand, vitamins help maintain microbial diversity and balance within the gut ecosystem [81,163,205,212].

For instance, vitamin B-12 supplementation has been linked to increased alpha-diversity in the adult gut microbiome, with a rise in *Faecalibacterium spp.* and a reduction in the proportion of the *Anaplasma phylum* [162]. Vitamin C supplementation can elevate levels of *Lachnospiraceae (Trichoderma spp.) and Blautia*, decrease *Anaplasma spp. and Enterococcus spp.*, and lead to favorable changes of inflammation and cognitive function [213]. Similarly, vitamin D supplementation enhances microbial diversity in healthy individuals, boosting *Bifidobacterium and Akkermansia*, lowering the abundance of pathogenic *Actinomycetaceae*, and reducing the F/B ratio [214]. It also increases populations of butyrate-producing bacteria (e.g. *Blautia and Roseburia*), enhances SCFA production, and lowers LPS-driven inflammation [215].

By ensuring an adequate vitamin supply, it may be possible to support the stability of the gut microbiota and mitigate secondary inflammation in patients with SCI, potentially contributing to better overall recovery and health outcomes.

##### Minerals

Zinc and selenium metabolism also rely on a balanced gut microbiota, and these minerals in turn influence microbial composition [[216], [217], [218]]. Striking the right balance is critical, as both deficiency and excess of these minerals can disrupt the intestinal microbiome. In mouse models, zinc deficiency leads to dysbiosis, inflammatory conditions, and compromised intestinal wall integrity [219], whereas zinc overload reduces microbial diversity, increases *Enterobacteriaceae* prevalence, lowers SCFA production, alters intestinal permeability, and triggers inflammation [27]. Clinical findings support similar outcomes in humans [220].

Likewise, selenium balance is important. Selenium deficiency raises levels of *E. coli, Clostridia, and Enterobacteria*, whereas selenium excess affects the abundance of *Turicibacter, Akkermansia*, and lactic acid bacteria, altering selenium status and related metabolites [26].

Calcium metabolism also depends on a healthy gut microbiota. By maintaining stable gut pH and SCFA levels, a balanced microbiome supports calcium absorption, influences calcium metabolism, and helps maintain bone density through modulation of serotonin levels [217]. Conversely, dysbiosis (e.g. *Oxalobacter formigenes*) can disrupt calcium metabolism and elevate the risk of kidney stones [221]—a particular concern for bedridden patients with SCI, who face a higher risk of calcium oxalate stone formation.

In short, maintaining appropriate micronutrient levels is essential for preserving gut microbiota balance and overall intestinal health after SCI.

Current evidence suggests that certain dietary patterns—such as following the MD, KD, or plant-based protein-rich diets, as well as practicing CR, supplementing with omega-3 PUFAs, and maintaining balanced vitamin and micronutrient intake—may significantly aid recovery from SCI. In contrast, prolonged consumption of high-fat or HFHS diets may harm patients. Although these findings provide a strong foundation for using specific nutrients and dietary patterns to influence gut microbiota in SCI, more long-term research and mechanistic studies are needed to fully understand these effects in SCI populations. Future large-scale, systematic investigations should focus on detailing how various dietary strategies interact with the gut microbiota in patients with SCI. Such work will pave the way for evidence-based, targeted nutritional support and rehabilitation protocols to optimize patient outcomes.

## Future Directions and Challenges

Nutritional interventions in SCI rehabilitation face several challenges due to individual differences in injuries, the complexity of long-term dietary interventions, and the intricate interactions within the “microbiota-gut-brain axis” and the “microbiota-gut-brain-spine axis.” Recent studies suggest a bidirectional regulatory relationship between SCI and gut microbiota. Introducing dietary factors adds more variables and possibilities to this system, but research on how diet, gut microbiota, and SCI interact is still limited. Future studies should address the following key challenges to facilitate the clinical application of dietary interventions in SCI rehabilitation.

First, the diversity among patients with SCI makes precise dietary interventions challenging. Variations in injury type, disease stage, neurological status, and metabolic needs limit the effectiveness of standardized approaches. Future studies should prioritize personalized nutritional plans that consider genetic background, gut microbiota composition, lifestyle, and functional status, with continuous monitoring to enhance long-term outcomes. Additionally, the heterogeneity of SCI significantly increases the complexity of nutritional intervention research. This complexity primarily arises from stage-specific pathophysiological characteristics and the dynamic temporal nature of nutritional needs. For example, the acute phase prioritizes neuroprotection, whereas the chronic-phase focuses on neural repair and the management of secondary complications. However, most studies on diet and the gut microbiota in SCI have concentrated on the chronic-phase, with limited attention given to the acute phase—primarily involving the KD. This research bias may result in a missed “golden window” for nutritional interventions during the acute phase and the erroneous extrapolation of chronic-phase findings to the acute stage, posing risks for clinical translation. Our review of research in this field aims to clarify current directions and underscore the urgent need for more studies that address SCI heterogeneity to bridge these critical gaps.

Secondly, the complex role of gut microbiota in SCI rehabilitation remains underexplored. Gut microbiota imbalance after SCI is closely linked to metabolic disorders, inflammation, and obstacles to neural repair. Diet, as a major regulatory factor of gut microbiota, influences neurological function and immune responses via the “microbiota-gut-brain axis” and the “microbiota-gut-brain-spine axis.” However, coordinating the interactions among diet, gut microbiota, and spinal cord repair is a critical challenge. Future research should investigate how different dietary patterns (e.g. KD, anti-inflammatory diet) affect neural recovery by modulating gut microbiota, and identify the specific mechanisms by which dietary interventions regulate the microbiota–gut–brain–spine axis.

Third, the feasibility and risk management of long-term dietary interventions are critical issues. Certain dietary patterns, such as high-protein and KD, may offer neuroprotective and metabolic benefits in the short-term but could lead to problems like bone metabolism abnormalities, cardiovascular risks, or gut microbiota imbalance with prolonged use. Future research should adopt multimodal intervention strategies that combine diet with exercise and medication and develop flexible dietary plans to balance therapeutic benefits and potential side effects.

Fourthly, the role of gut microbiota and dietary interventions in preventing and managing SCI-specific secondary complications—such as neurogenic bowel dysfunction, pressure injuries, autonomic dysreflexia, and abnormal lipid metabolism—remains insufficiently explored. Intestinal microbial imbalances may exacerbate bowel motility issues, systemic inflammation, and metabolic dysregulation, thereby contributing to these complications [20]. For instance, disrupted gut homeostasis could worsen abnormal lipid metabolism [222], whereas alterations in the gut–brain/spine axis can compromise autonomic stability relevant to dysreflexia [223]. Additionally, protein supplementation is complex and must be tailored to each patient's metabolic demands and clinical status [78]. Therefore, future research should investigate targeted dietary interventions that regulate the microbiota–gut–brain/spine axis. A deeper understanding of these mechanisms may pave the way for personalized nutritional strategies to mitigate secondary complications and improve long-term outcomes after SCI.

Finally, clinical translation and interdisciplinary collaboration are essential. Developing prerequired dietary plans requires integrating advances in neuroscience, nutrition, and microbiology, validated by large-scale, multicenter clinical trials to ensure safety and effectiveness. Establishing long-term follow-up mechanisms to evaluate intervention outcomes dynamically will help develop the best practices in SCI rehabilitation.

In summary, future research should focus on clarifying the bidirectional relationship among diet, gut microbiota, and SCI; developing personalized, multimodal intervention strategies; and advancing the efficient translation of basic research into clinical applications. This will provide patients with SCI with more precise and comprehensive rehabilitation pathways, ultimately improving their quality of life and long-term prognosis.

Building upon these insights, this review comprehensively explores the complex effects of dietary interventions on patients with SCI and their gut microbiome, highlighting their potential and necessity in improving patients’ long-term health and highlighting the key role of gut microbiome. Furthermore, we stressed the importance of personalized nutrition plans to address patient heterogeneity, optimize recovery outcomes, and prevent secondary complications.

The scientific significance of this study lies in delving into the complex relationships between diet, neurological repair, and metabolic regulation after SCI, providing a theoretical foundation for future clinical practice and research. Although challenges remain, such as determining optimal strategies for personalized nutrition and dietary-gut microbiota regulation, further exploration of dietary influences on the “microbiota-gut-brain axis” will aid in developing precise intervention strategies. In conclusion, this review offers new perspectives and recommendations for the long-term rehabilitation of patients with SCI, advancing the application of dietary interventions in neurological injury recovery and establishing a theoretical basis for comprehensive, multidisciplinary rehabilitation strategies.

## Author contributions

The authors’ responsibilities were as follows – ZL: designed research, drafted and revised the manuscript, and had primary responsibility for final content; XW, HD, WL, CZ: revised and edited the manuscript; ZT, XX, YP, JZ, HK: provided critical review; DY, FG, YY, YJ, JL: contributed to outlining the article and provided suggestions for revisions; and all authors: read and approved the final manuscript.

## Declaration of Generative AI and AI-assisted technologies in the writing process

During the preparation of this work, the author(s) used Open AI (Chat-GPT) to improve language and readability, with caution. After using this tool, the author(s) reviewed and edited the content as needed and take(s) full responsibility for the content of the publication.

## Funding

This work was supported by the Fundamental Research Funds for Central Public Welfare Research Institutes (2023CZ-2) and the Fundamental Research Funds for Central Public Welfare Research Institutes (2022CZ-3).

## Conflict of interest

The authors report no conflicts of interest.
